# Variability in the Integration of Peers in a Multi-site Digital Mental Health Innovation Project

**DOI:** 10.1007/s10488-023-01331-5

**Published:** 2024-01-21

**Authors:** Biblia S. Cha, Judith Borghouts, Elizabeth Eikey, Dana B. Mukamel, Stephen M. Schueller, Dara H. Sorkin, Nicole A. Stadnick, Xin Zhao, Kai Zheng, Margaret L. Schneider

**Affiliations:** 1 Department of General Internal Medicine, University of California, Irvine, Irvine, CA USA; 2 Herbert Wertheim School of Public Health and Human Longevity Science, University of California, San Diego, San Diego, CA USA; 3 The Design Lab, University of California, San Diego, San Diego, CA USA; 4 Department of Informatics, University of California, Irvine, Irvine, CA USA; 5 Department of Psychological Science, University of California, Irvine, Irvine, CA USA; 6 Department of Psychiatry, University of California, San Diego, La Jolla, CA USA; 7 Dissemination and Implementation Science Center, UC San Diego Altman Clinical and Translational Research Institute, La Jolla, CA USA; 8 Child and Adolescent Services Research Center, San Diego, CA USA; 9 Program in Public Health, University of California, Irvine, Irvine, CA USA; 10 School of Medicine, University of California, Irvine, 100 Theory, Suite 120, Irvine, CA 92697 USA

**Keywords:** Peer support, Mental health, mHealth, Mixed methods, Technology

## Abstract

Peer support specialists (“peers”) who have the lived experience of, and are in recovery from, mental health challenges are increasingly being integrated into mental health care as a reimbursable service across the US. This study describes the ways peers were integrated into Help@Hand, a multi-site innovation project that engaged peers throughout efforts to develop and offer digital mental health interventions across counties/cities (“sites”) in California. Using a mixed methods design, we collected quantitative data via quarterly online surveys, and qualitative data via semi-annual semi-structured phone interviews with key informants from Help@Hand sites. Quantitative data were summarized as descriptive findings and qualitative data from interviews were analyzed using rapid qualitative analysis methods. In the final analytic phase, interview quotes were used to illustrate the complex realities underlying quantitative responses. 117 quarterly surveys and 46 semi-annual interviews were completed by key informants from 14 sites between September 2020 and January 2023. Peers were integrated across diverse activities for support and implementation of digital mental health interventions, including development of training and educational materials (78.6% of sites), community outreach (64.3%), technology testing (85.7%), technology piloting (90.9%), digital literacy training (71.4%), device distribution (63.6%), technical assistance (72.7%), and cross-site collaboration (66.7%). Peer-engaged activities shifted over time, reflecting project phases. Peer-provided digital literacy training and technology-related support were key ingredients for project implementations. This study indicates the wide range of ways peers can be integrated into digital mental health intervention implementations. Considering contextual readiness for peer integration may enhance their engagement into programmatic activities.

## Introduction

Peer support specialists are individuals who have the lived experience of, and are in recovery from, mental health challenges (Fortuna et al., 2022a). Peer support specialists are increasingly being leveraged to produce better patient outcomes and have been identified by some as an indispensable element of mental health care (Fortuna et al., 2022b). While peer support specialists’ roles vary based on their context, key objectives of integrating them into mental health care include empowerment of patients in their recovery, sharing of their stories and journeys, advocating for destigmatization of mental illness, and providing education, counseling, and connections to resources (Bellamy et al., 2021). Peer support specialists can also provide patients and community members with validation of their experiences, a voice of lived experience to provide input to professional/medical teams, and positive examples and coping strategies for mental health challenges, stigma, and discrimination (Oborn et al., 2019). While there are mixed results on the impact of peer support on patient outcomes (Llloyd-Evans et al., 2014; Chinman et al., 2015; Oborn et al., 2019; Chien et al., 2019; Shalaby & Agyapong, 2020; White et al., 2020; Bellamy et al., 2021), the World Health Organization considers peer support an essential element of mental health recovery (Commission on Social Determinants of Health, 2008), and peer support specialists have been used globally to address mental health needs (Mpango et al., 2020). As of 2022, peer support specialists provide services that are reimbursable through Medicaid in 43 of 50 states across the US (Fortuna et al., 2022b).

While peer support specialists started off providing largely informal support either directly or via support groups, they are now more integrated into formal mental health service systems and provide formal support and trauma-informed care focused on recovery (Shalaby & Agyapong, 2020). Peer support specialists still engage in a range of activities, including case management, program development, evidence-based intervention delivery, coaching, mentoring, role-modeling and story-telling, as well as general support for recovery (Fortuna et al., 2022b; White et al., 2020). The settings in which they work also vary, and include inpatient, outpatient, and community-based contexts (Fortuna et al., 2022b). The shared thread in peer support specialist activities is their reliance on experiential knowledge delivered to others sharing a mental health challenge (Fortuna et al., 2022b). While peer support programs have also been developed for people with other health issues, such as cancer (Hoey et al., 2008) and diabetes (Warshaw et al., 2019), in this paper we focus specifically on peer support activities focused on mental health challenges.

### The Help@Hand Project

In 2017, California embarked on an innovative initiative called Help@Hand to understand whether and how digital mental health interventions (DMHI) can be integrated with the behavioral healthcare system to deliver a suite of DMHI to the broader community via county and city partners (Sorkin et al., 2022) to make DMHI more accessible across California’s large and diverse population. In recent years, there has been a particularly high interest in encouraging access to mental health services via online peer support communities (Merchant et al., 2022). This shift was accelerated with the onset of the COVID-19 pandemic, which was associated with both an increase in population-level mental health distress and an initial decrease in access to traditional in-person mental health services (Dong & Buey, 2020; Li et al., 2020; Blanchflower & Bryson, 2022). Digital peer support, which entails a wide range of peer support services facilitated via technology, has been found to be a promising approach to complement traditional mental health care (Fortuna et al., 2019). Digital peer support can include interventions that are delivered by peers via digital mental health applications, peer-to-peer connections on social media, chat applications, and other synchronous or asynchronous interactions with patients (Fortuna et al., 2020). A recent systematic review of digital peer support mental health interventions for people with serious mental illness concluded that there is a growing evidence base for the feasibility, acceptability, and effectiveness of such interventions (Fortuna et al., 2020). Furthermore, with the emergency declaration of the COVID-19 pandemic, digital peer support became reimbursable as a Medicaid service as many healthcare services switched to telehealth options (Fortuna et al., 2022a).

The Help@Hand project was funded as a 5-year “innovation project” through California Prop 63, commonly known as the Mental Health Services Act (California Department of Health Care Services, 2000). By definition, innovation projects are those that focus on novel approaches, strategies, or practices that contribute to learning, and are intended to increase access to services, service quality, or improve mental health outcomes, especially for underserved, unserved, and inappropriately served individuals (Mental Health Services Oversight & Accountability Commission, 2023). Administered by the California Mental Health Services Authority (CalMHSA), the Help@Hand project sought to integrate peer support specialists throughout its programmatic efforts, including the selection, testing, and piloting of DMHI, as well as the implementation and service delivery of DMHI.

Innovative components of the Help@Hand project included its leveraging of technology, its multi-site partners, and its emphasis on collaboration across participating counties and cities, which traditionally do not share resources or work together on project development or implementation. Twelve counties (Kern, Los Angeles, Marin, Modoc, Mono, Monterey, Orange, Riverside, San Francisco, San Mateo, Santa Barbara, and Tehama Counties) and two cities (Tri-City and City of Berkeley) across California joined together to learn and to implement innovative technologies. For ease of presentation, the counties and cities participating in Help@Hand will henceforth be referred to as sites.

The peer component of Help@Hand was ambitious in that it was envisioned as incorporating peers not only into service delivery but also into the co-creation of the program at every step, including the evaluation and selection of technologies, the tailoring of technologies to address the needs of specific populations, and the solicitation of feedback from the community to inform the process. Peers are rarely engaged as partners in co-creation of mental health-related programs and services (Åkerblom & Ness, 2023), so this innovative approach to increasing the accessibility of mental health technology offered a unique opportunity to document how peers contributed to co-creation across a variety of different public health contexts. In the context of the Help@Hand project, sites sought to integrate peers into the co-creation and implementation of various DMHIs, including diverse mental health apps, care coordination platforms connecting providers and clients, and peer-delivered chat applications (Help@Hand, 2022; Sorkin et al., 2022).

Help@Hand’s initiative to integrate peer support specialists (hereafter referred to as “peers”) into DMHI is the first of its kind to do so at the state level. Consistent with the intent of the innovation projects to promote learning that will inform efforts to increase access to mental health services, this study used a mixed methods design to document the ways that the peer component was implemented within the Help@Hand project. We addressed the following questions: (1) How did the various Help@Hand sites operationalize the peer workforce in terms of leadership structure and percent effort dedicated to the project as well as size of the peer workforce? (2) What contributions did peers make to efforts to plan and deliver DMHI to their diverse communities?

## Methods

### Study Context

California is the most populous state in the US, with an estimated 39 million residents across 58 counties as of 2022 (Perez et al., 2023). It is distinctively diverse, with more immigrants than any other state (27% immigrant population as of 2021) (Perez et al., 2023) and nearly half of the residents speak a language other than English at home (California Immigrant Data Portal, 2022). The 14 participating sites were spread across different parts of California, including urban, suburban, and rural regions ranging from large metropolitan areas with nearly 10 million residents to sites with less than 10,000 residents. Sites also were characterized by a wide range of resources, funding, and staffing capacity. Demographically, the sites in the Help@Hand project support approximately 19.4 million individuals that account for nearly half of California’s population (US Census Bureau, 2021).

### Study Participants

This evaluation project collected survey and interview data from individuals involved with and/or informed about the peer component of their respective sites’ Help@Hand projects. Within the scope of our study, all but two sites employed designated Peer Leads, who were peers who had some percentage of their time allocated to the Help@Hand project and assisted with the design, implementation, and integration of peers into their site's project. Peer Leads could share learning experiences, processes, resources, and products with other peer colleagues across the Help@Hand project during monthly peer-only calls. At sites without a Peer Lead, the Tech Lead (i.e., the overall Help@Hand project lead) or another designated key informant participated in surveys and interviews.

### Data Collection

The study was conducted by an evaluation team from the University of California, Irvine, using a mixed methods approach. Survey and interview data were collected in an iterative fashion from September 2020 to January 2023. This study was approved by the Institutional Review Board of the University of California, Irvine.

#### Survey Development

Survey items were constructed based on a rapid qualitative analysis of 22 semi-structured formative telephone interviews conducted in Winter and Spring 2020 with 14 Help@Hand key informants. Formative interviews were conducted and analyzed by the last author to identify themes around peer activities in support of the Help@Hand project. These themes then formed the basis of the forced-choice items included in the surveys, which consisted of questions regarding respondents’ roles within their respective sites, structure of the sites’ peer workforce, and activities in which peers had engaged during the prior three months.

In responding to the questions about peer activities posed in this evaluation study, key informants were asked to consider all peers engaged with Help@Hand, both peer support specialists hired in program management roles (i.e., Peer Leads) and peer networks with more narrowly delimited roles. For example, one site tapped into an existing workforce of persons with lived experience in mental health challenges to deliver counseling via a chat application, while another site recruited a group of adolescents with lived experience in mental health challenges to provide feedback on program elements. As the Help@Hand project moved into new phases and interviews identified new peer activities, these additional activities were incorporated into subsequent surveys. Specifically, items related to piloting DMHI were asked starting Winter 2021, providing technical assistance starting Spring 2021, and device distribution to community members starting Fall 2021.

#### Survey Data Collection

Surveys were administered quarterly starting in Summer 2020. Key informants from each site were emailed links to online surveys using REDCap (Research Electronic Data Capture), a secure, web-based software platform designed to support data capture for research studies hosted at University of California, Irvine (Harris et al., 2009, 2019). Up to six email reminders were sent every three days until surveys were completed. Surveys that were not completed within one month after the initial invitation were considered missing.

#### Interview Guide Development

Survey responses were used to inform bi-annual interviews, which were structured to elicit more in-depth information regarding each of the activities that was reported in the survey (such as outreach to the community, product testing, technical assistance, etc.). The open-ended nature of the questions allowed for dialogue and further probes of responses (See Appendix A for interview guide).

#### Interview Data Collection

Interviews were conducted in English and ranged from 30 min to 1 h. Interviews were initially conducted over the phone and later over Zoom with audio only. While interviews were not audio-recorded, the interviewer typed detailed, near-verbatim notes for the purpose of maintaining confidentiality and cultivating trust. Respondents were assured that responses would only be reported in aggregate or as anonymous quotes for illustrative purposes. Interviewers had extensive interview training, and one interviewer conducted all but one interview.

### Data Analysis

We used a mixed methods approach to analyze the data, wherein the core component of the analysis focused on descriptive survey results and qualitative analysis complemented quantitative findings (Morse & Niehaus, 2009). Quantitative descriptive findings were summarized as counts, frequencies, and indicators of central tendencies (i.e., means, medians). Means, percentages and ranges derived from the surveys provided indicators of the prevalence (percent of sites reporting an activity at least once) and consistency (mean percent of surveys over time on which an activity was reported) of each specific activity. Interviews were reviewed by the first and last authors to identify quotes that provided useful context or elaboration regarding activities. Throughout the data collection period, rapid qualitative analysis of interview notes was used to identify new activities for inclusion on subsequent surveys (Nevedal et al., 2021). In the final analytic phase, qualitative and quantitative results were brought together to allow the researcher team to interpret quantitative observations using qualitative insights.

## Results

From Summer 2020 to Fall 2022, quarterly surveys and semi-annual follow-up interviews documented peer activities within the Help@Hand project. Data were collected from a total of 117 surveys and 46 interviews (Table 1). One county employed two separate Peer Leads supporting two separate target populations throughout the entire project, so the number of interviews did not map precisely onto the number of active Help@Hand sites. In addition, three sites stopped participating in the Help@Hand project between Fall 2020 and Summer 2022. Overall, participation in the data collection was excellent, with eight sites completing 100% of the 10 surveys. One site only responded to the data collection in Summer 2020, after which they declined to provide further data. Nearly three-quarters of surveys were completed by Peer Leads (72%), 25% by Tech Leads, and 3% by other key informants. On average, the same individual completed 81% of the surveys and 83% of the interviews for that site. Surveys were completed by 23 unique individuals, and interviews by 19 unique individuals.

**Table 1:** Data collection and response rates between Summer 2020 and Fall 2022

Year (Help@Hand Year)	Time Period^a^	No. of Active Sites^b^	No. of Surveys (Response Rate)	No. of Interviews (Response Rate)
2020 (Year 2)	Summer	14	15 (100%)	13 (93%)
	Fall	14	13 (87%)	–
2021 (Year 3)	Winter	12	12 (92%)	12 (92%)
	Spring	12	11 (85%)	–
	Summer	12	12 (92%)	11 (85%)
	Fall	12	12 (92%)	–
2022 (Year 4)	Winter	12	12 (92%)	–
	Spring	12	10 (77%)	10 (77%)
	Summer	11	10 (83%)	–
	Fall	11	10 (83%)	–

^a^Time periods were defined as the following: Winter (Jan-March), Spring (April-June), Summer (July-Sept), Fall (Oct-Dec)

^b^Active sites were those remaining in the Help@Hand project

### Variability in the Peer Workforce Across Sites

In addressing our first research question regarding the person-effort dedicated to the peer component of Help@Hand, we examined the variability across the project sites. The most salient contrast was between those sites that identified a Peer Lead, signifying a commitment to engaging with the peer component of Help@Hand, and those that did not. Two sites never identified a Peer Lead during our data collection. One of them declined to provide data through surveys or interviews, but the other did participate through their Tech Lead, indicating at each survey that no peer activities had occurred.

Among the sites that did identify a Peer Lead responsible for the peer component of Help@Hand, the majority (at least 75% during Summer and Fall 2021) employed this individual full-time, although their percent effort devoted to the Help@Hand project varied widely. In the first round of surveys, 54% of the Peer Leads reported that less than 25% of their time was allocated to Help@Hand. Over time, the general trend was that about half of the sites allocated less than 50% of the Peer Lead’s time to Help@Hand.

In terms of the total number of peers engaged with the Help@Hand project at each site, there was considerable variability. One site was a clear outlier, as they had a robust peer workforce prior to joining Help@Hand and could assign peers to the project as needed. On the other hand, some sites did not employ any additional peers beyond the Peer Lead or did not have a Peer Lead. To account for this non-normal distribution, we computed the median number of peers across sites to examine patterns over time. The median number of peers engaged with Help@Hand ranged from 1.5 to 4.0 during the period of our data collection, with no clear pattern over time.

### Peer Activities

To address our second research question regarding the contributions that peers made to program efforts in planning and delivering DMHI to their diverse communities, we examined: (1) the different types of activities engaged in by peers, and (2) the total number of activities engaged in by peers.

#### Activity Types

Peers were engaged in a variety of activities across the Help@Hand project, demonstrating that they were able to adopt many different roles in the project. Table 2 shows the prevalence of the different peer activities (i.e., the proportion of sites that reported each activity at least once) and provides quotes from the interviews which demonstrate that their contributions were substantive and varied. Across the whole span of the data collection period, peers at more than 85% of sites were involved with creating educational materials (such as brochures, handouts, and websites), testing products, and piloting technology. Over 70% of sites reported peers being active in providing digital literacy training, and approximately two-thirds of sites reported that peers engaged in community outreach and providing technical assistance. Because a stated goal of the Help@Hand project was to leverage the transfer and sharing of information across the participating sites, we also asked about whether peers engaged in any cross-site collaboration. Overall, 57% of sites reported such collaboration at least once.

**Table 2:** Examples of peer activities as described in interviews with key informants

Activity	% sites	Illustrative Quotes from Interviews
Community Outreach	64.3	“Outreach is happening—I am doing it by emailing executive directors and directors of agencies that I am familiar with that would help us in serving their clients and reaching the community. The team has started doing canvassing—from the ground up. Tabling events at community events and canvassing—business to business to business in the community.”
Creating training and educational materials	85.7	“One peer that has been working on the peer chat came across some challenges—people asking for coping skills related to specific things that they were experiencing. She felt challenged by that because she was not familiar with it. She started researching and putting together lists of coping skills based on specific diagnoses. It is basically a resource guide for the other operators to use—to have suggestions of coping skills based on what the person is sharing that they are experiencing.”
Product Testing	85.7	“We had a list of about 20 different apps and we divided them up among the peers and then we came back to a table and reported our findings and which one we would want to keep on testing. We went down eliminating things that weren’t working and eventually got down to one.”
Piloting Technology	90.9	“The peer way is different in terms of thinking about the recovery process as incremental, using particular language. We had [the vendor] change the language around to give it a ‘real’ sound—there is peer influence on that app and working with that [vendor].”
Digital Literacy Training	71.4	“We have people who have been given smart phones by their children and they don’t know how to operate them. They might know how to make and receive calls, but nothing else. We started basic, showing them how to take pictures.”
Peers Receiving Digital Literacy Training	78.6	“[One of our peers] told me that a portion of the [digital literacy] training covered talks about being careful as peers about our ‘digital footprint,’ which included topics such as password safety, being careful about being ‘tagged’ in photos, and location on the phone.”
Technical Assistance	72.7	“Every Wednesday and Friday from 3–4 we have a Zoom office hour. People can come in and ask about using tech. Our peers will help support them through the Zoom platform.”
Device Distribution	63.6	“We had our tech distribution project and we had procured about 60 devices and we have given away about 50ish, still waiting for a couple of people to provide their onboarding packet. We partnered with 2 CBO’s [community-based organizations] that helped us distribute.”
Cross-County Collaboration	57.1	“We had a meeting with [site] and then recently we had a meeting with [site]. They wanted to hear from us—how did we deal with the distribution of the tablets, technology. We shared some documents as well. They are working with similar populations.”

#### Number of Activities

**Fig. 1:**
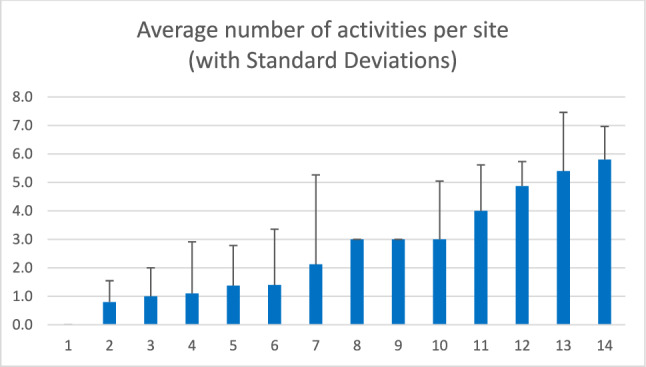
Average number of activities per site organized from least to most (with standard deviations)

The number of different activities in which the peer workforce was engaged at any one point in time also differed across sites (see Fig. 1). While some sites reported that the peer workforce engaged in an average of four or more activities over the course of data collection, others reported an average of less than one activity. The average number of activities provides a proxy measure of the extent to which a site integrated peers into Help@Hand projects. As illustrated in Fig. 1, the standard deviations in the number of peer activities were substantial at some sites. The dynamics behind these fluctuations in the number of peer activities differed by site. In a few sites, we observed a single quarter with a spike indicating a flurry of peer activity that was not subsequently sustained, whereas several sites experienced a general trend of increased peer activities over time and others reached a peak in 2021 and then reported a steady decline (quarter-specific data not shown).

It is worth noting that over time, the average number of peer activities reported across all active sites increased gradually; starting in Summer 2020, the quarterly averages were 1.75, 1.71, 2.64, 2.20, 2.50, 3.23, 3.00, 3.22, 3.78, and 3.50, respectively. Summer and Fall of 2020 had the lowest average number of activities, suggesting that the COVID-19 pandemic had a clear dampening effect on the peer component of the Help@Hand project. As restrictions on interpersonal contact were lifted and as peers gained greater familiarity with digital communication platforms, the number of activities increased until leveling out over the last year of data collection.

Through the interviews, key informants provided the context regarding the extent to which peers engaged in activities. For example, at one site reporting low peer engagement, the Peer Lead shared that they were excluded from local implementation-related process and communications, which limited peer engagement with related activities: “I would say that there is a challenge because there are people making decisions and I don’t know about them. I am getting it kind of third hand. I don’t know how involved peers were in any other aspect. Communication within the county –not great because it seems like we are launching it and I did not know.” On the other hand, a Peer Lead at another site which had high levels of peer engaged activities throughout the project reported, “Peer support specialists are fully invested and actively participating in every aspect of the project in terms of what the people in our community will be seeing or hearing from us as a team. They actually lead the team. They are involved in the brainstorming and decision-making processes. There is a peer in every single meeting except the Tech Lead meetings.”

#### Change over Time

The nature of the peer activities changed to some degree over time. As noted in the Methods section, the initial list of activities in the survey was derived based on qualitative interviews with the sites and additional activities were added as they emerged in later interviews. As described in more detail in the following sections, the peer activities described in the interviews provide additional information regarding the evolution of the Help@Hand project.

##### Materials Development and Community Outreach

**Fig. 2:**
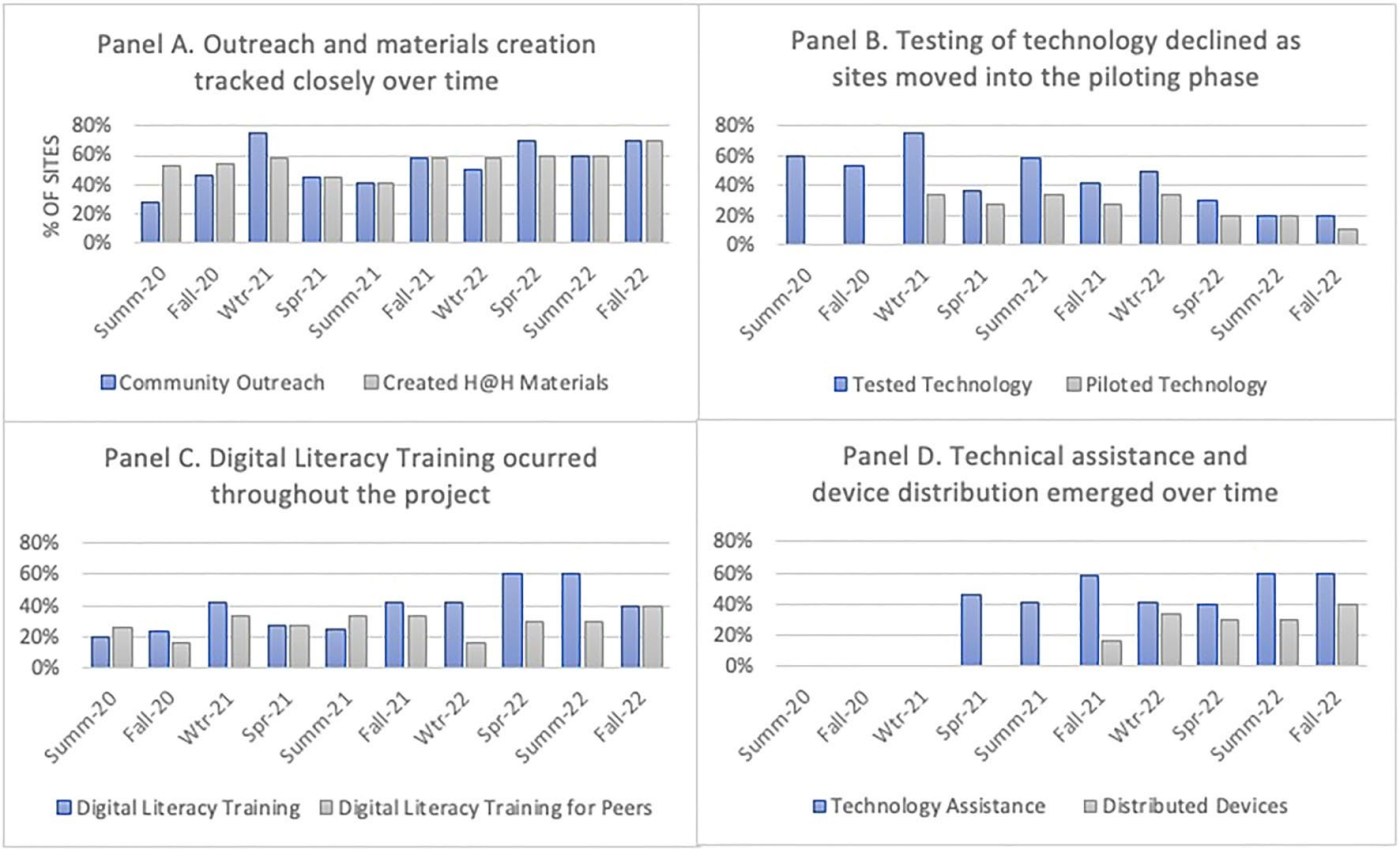
The nature of peer activities changed over time based on the stage of the Help@Hand project

Figure 2, Panel A, shows the percentage of sites in which peers were involved in creating materials for Help@Hand and in outreach over time. These two activities were closely linked, since most materials were created by the peers for them to use in their outreach efforts. During the first two quarters of survey data collection in Summer and Fall 2020, sites were restricted by the COVID-related stay-at-home orders that impacted their ability to engage in outreach, so it is not surprising that during this period they were more likely to be involved with creating materials than in reaching out to the community. As stated by one Peer Lead during the early days of the pandemic: “We haven’t been able to engage as much with our community with youth in the way that we want because given COVID the young people we have been working with have been inundated with being on Zoom calls and being on their phone and their computer all day so it didn’t feel right to ask them to join us for more Zoom calls to look at these apps. We have been struggling to engage with community.” Winter 2021 saw a spike in outreach, with 75% of the sites reporting that peers were engaging in outreach-related activities. While some sites continued to rely on digital forms for outreach, others were able to return to engaging in person: “We were very busy January through March [2021] because in addition to the “Get Appy” workshops monthly we also distributed 20 tablets and met with people in person for the first time in over a year. It was really different.” For the remainder of the data collection period, materials creation and outreach tracked closely with one another and increased over time. By Fall 2022, 70% of the sites reported that peers were creating materials and engaging in outreach.

##### Product Testing

Figure 2, Panel B, shows the change over time in the proportion of sites where peers were actively testing and/or piloting DMHI, which included apps as well as websites. Overall, technology testing, which involved both reviewing potential technologies and testing with site peers, was much more common in the earlier phases of the Help@Hand project (i.e., 54%–75% in the first three quarters of data collection) compared to the later phases of the project (i.e., 20–30% in the last three quarters of data collection). Technology testing was a necessary precursor to piloting a technology with site communities and was also something that peers could do while technology deployment was stalled as a result of COVID restrictions or contracting delays. The technology testing activities decreased over time as sites transitioned into the phase of piloting technologies. The approach to technology testing also varied across sites. One site described a very systematic approach that revolved around their transition-aged youth peers: “We would introduce the app on a Friday and get the youth all their registration materials and get them set up. Then we had assigned participant IDs so they could take a demographic survey. Then they would complete a technology survey … And then we also did a focus group at the end of the week-long period.” Another site chose to enlist a subcontractor to carry out the preliminary technology testing task with peer input: “We are working with … a consultant group. They went through all of [the apps] and highlighted some that met the requirements for our target population. We went through quite a few. Four of us went through all of them and we each came up with the top ten and then we had a discussion to narrow it down to the top 10 out of the 4 of us. Then we had the consultant gather more information about each one and then yesterday with that information that they gathered we narrowed it down to 3 or 4.” A third site took a less structured approach to engaging the peers in technology testing: “The peers are still working on the app, looking at it and making recommendations … They do it about an hour a week or so; 4 times a day for 15 minutes. That would be 4 peers out of my 5 peers.”

##### Piloting Technology

In Winter 2021, an item was added to the survey instrument to elicit information about whether peers were engaged in assisting sites with piloting the chosen digital mental health technology. About a third of sites reported that peers engaged in piloting DMHI during Winter 2021, and this rate stayed relatively steady through Winter 2022 (see Fig. 2, Panel B). Pilots were distinct from product testing in that they involved recruiting community members to use a given technology for a period of time and included structured data collection about users’ experience.

##### Digital Literacy Training

Digital literacy is defined as “the set of skills, knowledge and attitudes required to access, create, use, and evaluate digital information effectively, efficiently, and ethically” (Julien, 2018). Figure 2, Panel C, shows that digital literacy training (DLT) was delivered both to peers and to community members throughout the entire project. DLT included both foundational training focused on access and use of digital information, as well as specific training for how to use a site-specific DMHI. There was an overall increase in the proportion of sites delivering DLT to the community over time, with a peak occurring in Spring and Summer 2022, when 60% of sites reported that peers had been engaged in providing DLT. As sites pivoted from in-person to digital forms of outreach, the materials that were being created were often for supporting digital literacy education directed to the community. Digital literacy had been identified prior to the pandemic as a necessary component of technology uptake, but the necessity of interacting with the community remotely accelerated attention to the community gaps in this area. As explained by one Peer Lead: “We have our peers refining the digital mental health literacy curriculum … We did that because … we are trying to introduce people to apps, privacy, consents; all of those things that may help them have a different level of intake when we do start the pilots.” The subsequent decline in DLT in the final quarter of data collection is likely reflective of some sites ramping down their efforts in anticipation of the ending of the Help@Hand project and others diverting their peer efforts into technology piloting.

##### Peers Receiving DLT

 It is important to note that 11 sites (78.6%) reported that peers themselves also received DLT at some point, including over 40% of sites in any given quarter (see Fig. 2, Panel C). In the interviews, sites reported the peers themselves needed to acquire knowledge about the technology before they could effectively deliver DLT to their communities. One Peer Lead reported, “I am doing all these remote focus groups—it is a learning curve even for me. What I learned is that you really have to know what you are doing, because every situation is different technologically, what internet browser they are using, what device they are using. You really have to know it all to get them able to use the technology.”

##### Technical Assistance

In Spring 2021, an item was added to the survey instrument to ask about whether the sites were providing technical assistance in support of DMHI to the community. Figure 2, Panel D, shows the proportion of sites reporting that they were providing such assistance over time. Technology assistance involved troubleshooting for the specific DMHI interventions which sites had chosen and were delivering to their communities. The proportion of the sites providing technical assistance reached 60% at the end of data collection, while 66.7% of the sites reported that peers provided technical assistance at some point across all quarters. Sites varied in how peers provided technical support. One site utilized the peers as a go-between to facilitate community members’ enrollment in a technology-based counseling service: “Usually the clinic staff will communicate with the [vendor] team and those people get an email about someone who is going to be onboarded. [The peers] follow up with that person. Offer a phone, offer to show how to load the app and register … then they hand it to the care team. This is usually done in person. We prefer to have that face-to-face contact. Our people really love that.” Another site described an approach to providing technological assistance that was need-driven: “Our office will field any request regarding Help@Hand. When we advertise county-wide, we have an email address, and anyone can send us a message and we get all kinds of requests. In-service on kiosks, walkthrough of an app, [request to] co-facilitate Get Appy at Transition-aged Youth centers. It varies.”

##### Device Distribution

 In Fall 2021, an item was added to the survey instrument to ask about peers being involved in device distribution (see Fig. 2, Panel D). Although not part of the original vision for Help@Hand, device distribution emerged organically as the participating sites realized that access to hardware (cell phones, tablets) with the right specifications to support DMHI was a substantial barrier for community members. By the end of our data collection period, 63.6% of the sites had reported at least once that peers were active in distributing devices. As explained by one Peer Lead, this activity required considerable involvement from the peers: “We were happy to give out the tablets. We did not want to just hand people a box, we really wanted them to know how to do it. It took a 2-hour orientation outdoors, so some of them were very cold, but we did the best we could, and we are happy that people are using the tablets.”

##### Cross-Site Collaboration

Cross-site collaboration was very common throughout the Help@Hand project, with two-thirds of sites (67%) reporting cross-site collaboration at least once across all time periods. The collaboration activities took many forms. Some peers reported direct contact with other Help@Hand sites through which they learned from their colleagues’ experience: “We talked to [another site], who went through their tech distribution program and they had a lot of insights. They work with older adults, and they did not anticipate that they would need as much tech support as they did.” Other examples of cross-site collaboration involved one site making materials available to other Help@Hand sites: “We join the Peer calls every month and people have come to us because we have already distributed tablets so we created documents and we have sort of like a scrapbook of how we distributed tablets … we shared [them] on the CalMHSA google documents for the team. Anyone can go there and see the forms that we have used, how did our training with them in person. We said please use our documents.” Peers also reported examples of cross-site collaboration in terms of transferring excess resources, such as sharing application licenses or surplus devices.

## Discussion

By combining periodic surveys with interviews, this mixed methods study obtained a rich perspective on how peers were engaged in an effort to leverage DMHI to improve access to mental health services across multiple sites in California. Our analysis of how peer integration was operationalized in the Help@Hand projects provides an important and timely case study for sites considering integrating peers into county- or city-wide DMHI projects. Peers have been established as a part of community-facing mental health programs both across the US (Bellamy et al., 2021) and internationally (Charles et al., 2021; Stratford et al., 2019), contributing to the recent surge of mental health services provided via online support (Merchant et al., 2022). However, our study points to a far wider range of roles that they can play in a DMHI. Our findings provide initial information about what kinds of project activities peers can engage in as part of local efforts to leverage DMHI to increase accessibility to mental health services. By documenting these efforts in the California Help@Hand project, our study provides useful insights into the potential for integrating peers across diverse contexts and can help guide expectations of other sites considering investing in such efforts.

### Varied and Shifting Roles

This years-long project provided important snapshots of how the peer workforce contributed to varying roles in their respective programs. At some sites, peers’ voices were sought and integrated into nearly every step of the Help@Hand project, from providing input on community-facing marketing materials and products to choosing and testing DMHI products, piloting the chosen technologies, distributing devices, and personally providing DLT and technical assistance to community members. Peers brought their lived experience into informing their strategies on how to reach target communities when providing feedback about how to best tailor and market the DMHI projects at their respective sites. The broad integration of peers into Help@Hand provides support to research indicating that peers can be helpful in reaching diverse populations, including those who are traditionally harder to connect to mental health services (Bellamy et al., 2021). While much of the existing literature has investigated the impact of directly providing peer support on consumers’ well-being and mental health (Fortuna et al., 2022b; Mutschler et al., 2022), and the types of organizational support that facilitate successful peer support (Zeng & McNamara, 2021), this study indicates that peers are also valuable in non-consumer facing programmatic capacities such as materials development and technology testing.

Our study also made it clear that peer activities changed with the shifting phases of the Help@Hand project at various sites. While there were certain activities, such as community outreach, that were maintained consistently throughout the project at most sites, peers focused their time and attention to different activities based on needs and timelines. For example, testing products and piloting technologies largely occurred earlier in the project, while technology assistance and device distribution increased over time as the project progressed.

### Sites Varied in Their Levels of Peer Engagement in Help@Hand Activities

The finding that the number of different activities in which the peer workforce participated varied across sites and over time suggests that there were contextual factors influencing how many and what kind of activities peers had the opportunity to support. One contextual factor was the pre-existing peer structure at each site. As noted, in regard to the size of the peer workforce, one site entered the project with no peer workforce and maintained this status throughout, whereas another site brought to the project a pre-existing peer workforce that was robust and sustained. Other sites fell in between these two extremes. The extent to which sites had prior capacity for and experience with integrating peers into mental health services may have been a factor in the number and range of activities supported by peers at each site. Similarly, a recent systematic review of the peer implementation literature concluded that having peers as an integrated component of services, rather than as an add-on feature, facilitates programmatic implementation of peers (Mutschler et al., 2022).

Another contextual factor that likely impacted the degree to which each site was able to involve peers in Help@Hand activities was the percent effort of the Peer Lead that was dedicated to the project. At any one time, approximately half of the Peer Leads reported having less than 25% of their time allocated to the Help@Hand project, but four sites employed full-time Peer Leads to support the project, which likely afforded greater integration of peers into multiple activities at those sites. This finding aligns with prior research indicating that adequate funding and time is a key factor for peer implementation (Mutschler et al., 2022).

In terms of the changes over time in the number of activities involving peers, some of these fluctuations were clearly related to the COVID-19 pandemic and others were likely a function of site-specific conditions. Starting in Winter 2021, the average number of peer workforce activities reported across the Help@Hand projects moved toward a number between 3 and 4 and remained stable at that level for the last year of data collection. Possibly, this finding suggests an optimal range of the number of activities that the peer workforce can be involved with at any one time in such a program. Anecdotal evidence from the interviews suggests that the participating sites attempted to strike a balance between limiting the pressure on peers associated with multi-tasking and meeting program goals of integrating peers throughout the program.

### Peers Offer a Pathway to Collaboration

Our study also highlights the potential for peers to act as a communication bridge between multiple sites simultaneously engaged in parallel interventions. The regular monthly Peer Lead-only calls provided a forum through which peers could interact regularly to share resources, tools, ideas, and even surplus licenses and devices. Peers learned from others’ experiences on a range of issues, exchanging information about tailoring community outreach, DLT, and piloting and implementation of specific technology, contributing to the stated goal of transfer and sharing of information across the participating sites. Scholars have previously highlighted that collaborative peer learning experiences, including virtual ones, can enhance peer experiences and help them to work together to address context-specific issues (Cronise, 2016).

A distinctive feature of the Help@Hand project was the diversity of its sites, which included urban, suburban, and rural regions; large metropolitan areas with millions of residents to sites with just a few thousand residents; as well as a wide range of available resources, funding, and staffing capacity. Peers represented one channel of communication across the Help@Hand sites that facilitated information and resource exchange.

### Peers Deliver Technology-Related Training and Support

Finally, while the stated goals of the Help@Hand project were to make DMHI more accessible to California’s diverse population, it was clear early in the project that the baseline digital literacy of the target communities was insufficient for their members to effectively use DMHI, resulting in over 70% of sites providing DLT and/or technology assistance as part of their projects. While needs vary, our findings show that some of the populations with the most unmet mental health needs may also be those with low digital literacy levels, or who need the most intensive technological assistance, such as the elderly (Wang et al., 2007), people with lower income (Yang et al., 2019), those with poorer health (Corscadden et al., 2019) and ethnocultural minorities (Urbanoski et al., 2017).

### Implications

Our study described the innovative efforts to integrate peers into a multi-site state-wide project throughout the project planning, development, and implementation of DMHI to meet community needs. Given increasing numbers of state-wide policies across the US that allow peers to be reimbursable by Medicaid and the newness of such efforts in California, we suspect there are many sites interested in learning how to partner with the peer workforce. Our study is an opportunity for them to consider the implications of integrating the peer workforce into efforts to implement DMHI.

First, the variety of peer activities and their changing nature over time indicate that sites who desire to integrate peers should plan for diverse and shifting responsibilities through project phases. While some sites had multiple peers dedicated to different project activities, anticipating that peers may engage in a wide array of activities that may shift as the project progresses has implications for the hiring and retention of peers. For example, existing scholarship indicates a lack of clarity in peer roles and responsibilities hinders peers’ emotional well-being, which may contribute to staff turnover (Debyser et al., 2019). Furthermore, there are specific implications for the recruitment and hiring of peers for DMHI projects which may require peers to have some familiarity with and ability to navigate and even teach others about technology, both hardware and software. In the future, counties or cities seeking to integrate peers into a program featuring technology may want to hire peers with a technological background or plan to provide DLT.

In addition, the Help@Hand project’s collaborative peer calls were a meaningful and resourceful way for Peer Leads to connect, share, and learn about resources and new initiatives from each other. While scholars have called for more systematic collaboration with other professionals in the mental health system (Mirbahaeddin & Chreim, 2022), providing space and time for peers to collaborate with one another may be another meaningful way to optimize their participation in DMHI planning and implementation.

Finally, our study also indicates that sites considering DMHI implementation should first recognize that equipping communities with digital literacy is a necessary first step to making DMHI accessible and relevant. These findings align with existing studies which have found that insufficient digital literacy poses a barrier to older, younger, as well as socioeconomically and geographically disadvantaged groups (Kemp et al., 2021) who may want to use digital avenues for accessing healthcare, and that patients interested in using digital platforms may require support, training and technical assistance to access them (Camacho & Touros, 2023; Hernandez-Ramos et al., 2021). As a result, budgeting, funding, and timelines for planning and implementation should realistically include steps to address community needs for this type of support, particularly the support that can be delivered by peer support specialists. Partnering with other organizations which already provide DLT to tailor materials for respective communities is yet another aspect to consider when seeking to provide DMHI to diverse communities.

### Limitations

There are several limitations in this study. First, while we sought to conduct surveys and interviews with all Help@Hand Peer Leads, some sites with limited peer activity did not respond to surveys or interviews consistently, which may have led to results biased towards reporting activities undertaken at responsive sites (who may have been the most active). A related limitation is that the binary forced-choice nature of survey responses may be subject to reliability issues related to intracategory variability, in which respondents may have had diverse interpretations of what constitutes an activity (Dohrenwend, 2006). However, the reported range of activities in which peers engaged provides examples to other sites who may be considering integrating peers into DMHI projects. Second, staff turnover contributed to some missing data, as sites did not always have a Peer Lead or other key informant identified to respond to surveys and interviews and to potential variability in respondents’ interpretations of the survey questions. However, 81% of surveys and 83% of the interviews were completed by the same individual across sites, suggesting that the impact of variable interpretations may have been modest. Third, this study reports aggregate percentages of activities undertaken by peers. Future studies may provide case studies of specific sites to show a detailed picture of what factors guided their processes, products, and outcomes. Fourth, our study used detailed, near-verbatim notes of interviews rather than transcribed recordings. However, we believe that by assuring respondents’ anonymity, these practices fostered trust and led them to share with a higher degree of openness and honesty that would have been unlikely if recorded. The rigorous attention to peers’ privacy also led to the decision not to track peer demographics, so we are unable to report on what personal characteristics (e.g., age, language) peers may have shared with the target populations at their site. Furthermore, only one person was initially engaged with identifying themes from the initial interviews, which may have contributed to biases. However, the iterative process of collecting both survey and interview data provided an ongoing means of checking the validity of relevant themes.

## Conclusion

Our study included 14 counties and cities across California and described the variability of their efforts to integrate peers to make DMHI more accessible to their respective communities. We found that peers engaged in a wide range of activities that shifted over time, including community outreach, DMHI testing and piloting, and facilitating DLT and technology support. However, the degree of peer engagement with Help@Hand activities varied by site. Our analysis of how peer integration was operationalized in the Help@Hand effort provides important and timely information for other sites who plan to integrate peers into DMHI projects. This evidence is especially salient given peer support services are now a reimbursable service for Medicaid in most US states.

## Appendix A: Interview Guide

Thank you for taking the time to talk with me again. I would like to remind you that I will not share your individual responses with anyone. We will only report and share what we learn from you after it has been combined with information from other counties/cities. There are no right or wrong answers. You are the expert. We are here to understand your perspectives and experiences. Do you have any questions before we begin?

Great! Let’s start with some general questions about your county’s/city’s utilization of Peers to assist with onboarding clients or users to Help@Hand. For reference, these questions refer to the time period that includes (previous three months).

A. “I am going to ask you some specific questions that build on the information you provided in the survey, but before I do that, I would just like to get a sense overall of how things are going in your (county/city), especially with regards to the Peer component of Help@Hand. Can you give me an overall assessment?”
B. “In your survey, you indicated that during the (quarter) of (year) the Peers :
C. “Your survey also indicated that (county/city) experienced the following challenges to involving Peers in Help@Hand during this time period:
D. “You also reported that (county/city) has had the following successes that have resulted from the Peer component of Help@Hand.
E. “What changes, if any, would you recommend for the Peer component of H@H moving forward?”
F. “How effective do you think the Peers are currently in supporting Help@Hand?” Why or why not?
G. “Have you had a chance to look at the (Year) Evaluation Report produced by the team at UCI?”
  a. If so, how, if at all, did it inform your Help@Hand programming?

- Anything else?
